# IL-18-based combinatorial adjuvants promote the NK-DC-mediated production of the CCR7 ligand CCL19 in lymph nodes from cancer patients

**DOI:** 10.1186/2051-1426-1-S1-O21

**Published:** 2013-11-07

**Authors:** Jeffrey L Wong, Ravikumar Muthuswamy, David L Bartlett, Pawel Kalinski

**Affiliations:** 1 Department of Surgery, University of Pittsburgh School of Medicine, Pittsburgh, PA, USA; 2 University of Pittsburgh Cancer Institute, Pittsburgh, PA, USA

## 

Effective accumulation and interaction of mature dendritic cells (DCs) and naïve T cells within lymph nodes (LNs), driven by the CCR7-CCL19/CCL21 axis, are critical for the induction of adaptive T cell immunity. Human natural killer (NK) cells activated by IL-18 exhibit unique 'helper' activity in promoting productive DC-T cell interactions, inducing dendritic cell (DC) maturation and the type-1-polarization of DC-primed T cell responses. Here we demonstrate that such IL-18-induced 'helper' NK cells uniquely induce high DC production of CCL19 in a TNFα and IFNγ-mediated mechanism, dependent on secondary NK cell stimulation with the additional inflammatory signals IFNα, IL-15, IL-12, or IL-2. Helper NK cell-activated DCs promote efficient CCR7-mediated recruitment of naïve CD8+ T cells, and subsequently induce their expansion and acquisition of granzyme B. Using an ex vivo explant culture system of LNs isolated from colorectal cancer patients, we further demonstrate enhanced expression of CCL19 in human tumor-associated lymphoid tissue induced by treatment with helper NK cell-stimulating factors. Our data indicate the ability of two-signal-activated 'helper' NK cells to promote LN production of the DC- and naïve/memory T cell-attracting chemokine CCL19, and provide rationale for therapeutic application of IL-18-containing 'combinatorial adjuvants' to promote the induction of anti-tumor immunity.

